# Folate and Its Significance in Depressive Disorders and Suicidality: A Comprehensive Narrative Review

**DOI:** 10.3390/nu15173859

**Published:** 2023-09-04

**Authors:** Timur Liwinski, Undine E. Lang

**Affiliations:** Clinic for Adult Psychiatry, University Psychiatric Clinics, University of Basel, Wilhelm Klein-Strasse 27, CH-4002 Basel, Switzerland; undine.lang@upk.ch

**Keywords:** mental health, folic acid, folate, mood disorders, depression, suicide, suicidality, nutraceuticals

## Abstract

Depressive disorders pose significant challenges to global public health, necessitating effective prevention and management strategies. Notably, the occurrence of suicide frequently coincides with depressive episodes. Suicide is as a paramount global health concern that demands efficacious preventive strategies. Current psychiatric approaches heavily rely on pharmacological interventions but have had limited success in addressing the global burden of mental health issues. Suboptimal nutrition, with its impact on the neuroendocrine system, has been implicated in the underlying pathology of depressive disorders. Folate, a group of water-soluble compounds, plays a crucial role in various central nervous system functions. Depressed individuals often exhibit low levels of serum and red blood cell folate. Multiple studies and systematic reviews have investigated the efficacy of folic acid and its derivative, L-methylfolate, which can cross the blood–brain barrier, as stand-alone or adjunct therapies for depression. Although findings have been mixed, the available evidence generally supports the use of these compounds in depressed individuals. Recent studies have established links between the one-carbon cycle, folate–homocysteine balance, immune system function, glutamate excitation via NMDA (N-methyl-D-aspartate) receptors, and gut microbiome eubiosis in mood regulation. These findings provide insights into the complex neurobiological mechanisms underlying the effects of folate and related compounds in depression. Through a comprehensive review of the existing literature, this study aims to advance our understanding of the therapeutic potential of folic acid and related compounds in depression treatment. It also seeks to explore their role in addressing suicidal tendencies and shed light on the neurobiological mechanisms involved, leveraging the latest discoveries in depression research.

## 1. Introduction

Depressive disorders are prevalent mental illnesses characterized by long-lasting periods of depressed mood and diminished interest or pleasure [1]. According to the Diagnostic and Statistical Manual of Mental Disorders, Fifth Edition (DSM-5), major depressive disorder (MDD) is characterized by symptoms such as mood changes, anhedonia (loss of interest or pleasure), decreased energy levels, changes in appetite and weight, and feelings of worthlessness that persist for most days over a period of two weeks or more [2]. Depression stands as a significant public health concern and holds the distinction of being the foremost contributor to worldwide disability [3]. In Europe, the 12-month prevalence of depression is reported to be 6.9% [4]. Depression significantly affects various aspects of life, including relationships, academic or occupational performance, and overall wellbeing. Individuals who have experienced abuse, severe losses, or other stressful events are more susceptible to depression [5]. Women are more affected by depression than men with a 50% higher prevalence [6]. Globally, an estimated 5% of adults suffer from depression, equating to approximately 280 million individuals [7]. Depression is strongly associated with suicide, which claims the lives of over 700,000 people annually, making it the fourth leading cause of death among individuals aged 15–29 years [1]. The prevention and management of depressive disorders have become a paramount global public health priority [8]. The impact of depressive disorders extends beyond individual suffering, affecting families, communities, and societies at large [9,10,11]. By addressing these disorders through effective prevention strategies and comprehensive management approaches, we can alleviate the substantial burdens they impose, improve overall wellbeing, and enhance the quality of life for individuals and populations worldwide [12]. Despite the availability of effective treatments for depression, the majority of individuals in low- and middle-income countries do not receive adequate care due to various barriers, including insufficient investment in mental health care, a scarcity of trained healthcare providers, and the presence of societal stigma associated with mental disorders [13]. Individuals affected by depression face significant medical consequences and associated complications [14]. While depression typically follows an episodic pattern, it transitions into a chronic condition in approximately 15–25% of individuals affected by the disorder [15].

Guidelines, such as those provided by the National Institute for Health and Care Excellence in the UK, emphasize a holistic biopsychosocial approach to depression treatment, and evidence indicates that psychological interventions, social support, and exercise play significant roles [16]. However, in cases of moderate or severe depression, medication treatment is often deemed necessary [16,17]. The STAR*D trial (Sequenced Treatment Alternatives to Relieve Depression), the largest study conducted on depression treatment, took place in the United States with around 4000 participants. The trial aimed to explore various treatment alternatives for depression. The results from the trial revealed that only 37% of participants achieved remission following a 12-week treatment trial with citalopram. Subsequently, after four treatment trials, only 67% of participants achieved remission. These findings underscore the challenges associated with achieving optimal treatment outcomes in depression and highlight the need for further research and alternative therapeutic approaches [18]. Due to the high rate of non-response to standard SSRI (selective serotonin reuptake inhibitors)/SNRI (serotonin and norepinephrine reuptake inhibitors) monotherapy, clinicians face the dilemma of determining the next course of action. Options include increasing the dosage, switching to a different antidepressant, or augmenting treatment with psychotropic or antipsychotic medications. However, these approaches may lead to unwanted side effects and may still prove ineffective in alleviating patient symptoms. Consequently, there is a pressing need for safer and more effective alternatives to address treatment-resistant depression [19,20]. One potential strategy for addressing inadequate response to standard antidepressants is the augmentation of treatment with nutraceuticals, which are pharmaceutical-grade nutrients [21]. Among them, folic acid, or its active form, L-methylfolate, shows promise due to folate’s association with an elevated risk of depression, severe depressive symptoms, prolonged duration of depressive episodes, and increased likelihood of relapse [22,23]. The link between inadequate diet and the risk of depression is well-established [24]. Presently, there is an increasing interest in enhancing diets or utilizing particular dietary components to optimize outcomes in depression treatment [25]. Depressed individuals frequently manifest diminished levels of serum and red blood cell folate [26]. This deficiency is of considerable importance, as reduced folate levels could potentially hinder the responsiveness to antidepressants [22] and even lithium treatment [27]. Therefore, a comprehensive understanding of the mechanistic underpinnings of folate’s actions is warranted [28]. Furthermore, establishing its effectiveness among depressed individuals exhibiting different levels of folate deficiency, as well as those without deficiency, is of pivotal importance. Equally vital is the need to enhance psychiatrists’ awareness concerning the central significance of folate status in this particular context. By incorporating folate nutraceuticals into depression treatment, it is possible to provide a potential solution for individuals who do not respond adequately to standard antidepressant therapy [22,23,26,29,30,31,32].

By synthesizing and analyzing the available literature, this review aims to provide a comprehensive understanding of the therapeutic potential of folic acid and related compounds in treating depression and their role in addressing suicidal tendencies. It also seeks to shed light on the neurobiological mechanisms that may underlie their effects, incorporating recent discoveries and advancements in the field of depression research. This review predominantly centers on the literature addressing depressed adult women and men within non-elderly global populations. However, the insights it provides may also hold relevance for pediatric and geriatric populations.

## 2. Method

For the identification of pertinent studies within this narrative review, we executed a comprehensive literature search. We selected the CENTRAL, MEDLINE, and Embase databases as recommended by the Cochrane Handbook, which underscores their reputation for providing comprehensive coverage of the biomedical literature while maintaining stringent quality standards [33]. This approach was consciously chosen to provide a qualitative synthesis of diverse research findings in a complex field. The rationale behind opting for a narrative review, rather than a systematic one, rests on the distinct nature of our investigative aims. We sought to offer a holistic exploration of the interplay between folic acid, L-methylfolate, and depression, encompassing various dimensions that impact treatment responses. The search strategy entailed employing Boolean operators to combine a range of pertinent terms such as “folic acid”, “folate”, “L-methylfolate”, “depression”, “microbiome”, “probiotics”, and numerous others. This dynamic amalgamation of terms allowed for a comprehensive survey of the literature. We meticulously navigated the interplay of these terms through Boolean operators (e.g., AND, OR) to ensure thorough coverage. Our primary focus was on individuals who fell within the non-elderly age group, encompassing both women and men. Within this demographic, our investigation primarily targeted cases of primary unipolar or bipolar depression. We deliberately excluded studies where depression was a secondary phenomenon stemming from conditions such as neurodegenerative diseases. However, it is important to note that while our primary emphasis was on the specified population, the insights gleaned from our findings could potentially hold relevance for broader populations as well. Solely English-language publications were considered in this review. In tandem with the electronic database search, a meticulous manual cross-literature survey was conducted. Those articles deemed relevant in this initial screen were retrieved in full text for in-depth evaluation. Notably, the narrative review method was preferred to provide a contextual and nuanced synthesis of findings, given the intricate and multifaceted nature of the subject matter. Through this narrative approach, we aimed to holistically analyze and amalgamate insights garnered from the array of selected studies. This method allowed us to discern patterns, associations, and implications beyond the confines of a systematic review’s methodology. In our synthesis, data were methodically extracted from the selected studies and distilled into a comprehensive overview, accentuating key facets of the topic’s landscape. The assessment of study quality and relevance was carried out based on criteria encompassing the study design, sample size, methodology, and the soundness of outcomes.

### 2.1. The Relationship between Depression and Nutritional Deficiencies

Psychiatry is currently facing a significant moment where the prevailing model, which primarily relies on pharmacological interventions, has only provided limited improvements in addressing the global burden of mental health issues [34]. Recognizing the complexity of mental health determinants, there is an increasing body of compelling evidence highlighting the critical role of nutrition in the prevalence and development of mental disorders. This suggests that diet is just as crucial to psychiatry as it is to other medical specialties such as cardiology, endocrinology, and gastroenterology [35]. This recognition underscores the importance of considering nutritional factors in the context of mental health and expanding the scope of psychiatric practice to incorporate dietary interventions [36]. There is growing evidence suggesting that suboptimal nutrition plays a role in the development and progression of behavioral health disorders, and it can also hinder the effectiveness of treatment and recovery [37]. The underlying pathology of depression may be influenced by suboptimal nutrition due to the crucial role that nutrients play in the functioning of the neuroendocrine system. Various nutrients, such as tryptophan, vitamin B6, vitamin B12, folic acid (folate), phenylalanine, tyrosine, histidine, choline, and glutamic acid, are essential for the synthesis of neurotransmitters like serotonin, dopamine, and norepinephrine [37]. These neurotransmitters are involved in regulating mood, appetite, and cognition, thus highlighting the importance of adequate nutrient intake for optimal mental health [38,39,40]. The International Society for Nutritional Psychiatry Research has advocated for the integration of nutritional medicine into mainstream psychiatric practice, emphasizing the need for research, education, policy, and health promotion to support this innovative approach [38]. However, the implementation of this framework faces challenges due to the complexity and multidimensional nature of both mental health and nutrition [41]. Depressive disorders are multifactorial and do not have a singular cause. Various factors contribute to an increased risk of developing depression, including sex, gender, socioeconomic status, social support, levels of stress, alcohol and drug use, genetic and epigenetic influences, inflammation, the presence of medical illnesses, endothelial dysfunction, and dietary patterns [42]. These factors interact and influence each other, creating a complex web of risk factors that can contribute to the development and progression of depressive disorders. Determining the exact contributions of individual factors in the relationship between diet and mental health is challenging, and analyses may be affected by residual confounding. The nature of studying diet and behavioral health disorders epidemiologically is prone to reverse causation, where poor diet can be both a cause and a consequence of these disorders, indicating a bidirectional relationship [37]. Begdache et al. highlighted the intricate interplay between mental wellbeing and healthy lifestyle practices, where positive reinforcement exists between them. A lack of healthy lifestyle practices can lead to decreased mental wellbeing, which further diminishes the adoption of healthy habits, creating a cyclic pattern [43]. Numerous clinical trials have investigated the impact of dietary pattern modifications on depressive symptoms. The latest systematic review and meta-analysis, comprising 16 randomized controlled trials (RCTs) with a total of 45,826 non-clinically depressed participants, found that interventions based on whole-dietary interventions led to a reduction in depressive symptoms compared to control conditions, whether active or nonactive [44]. Likewise, a systematic review of RCTs focused on predominantly nondepressed participants and examined interventions utilizing a whole-diet approach. The review found that out of 17 studies, 8 reported significant improvements in depression scores compared to the control group. The effect sizes observed ranged from small to very large [45]. In addition to overall dietary patterns, deficiencies or imbalances of specific micronutrients can significantly impact mood and mental wellbeing [46]. Micronutrients play a crucial role in the metabolic pathways that contribute to the development and proper functioning of the nervous system. Insufficient intake of these micronutrients can have a negative impact on psychological wellbeing, increasing the risk of depressive disorders. Some micronutrients that are closely linked to mental health include folic acid, vitamin B6, vitamin B12, vitamin D, zinc, and magnesium. Insufficient intake of multiple micronutrients, amounting to a deficiency in four or more essential micronutrients, significantly increases the risk for depression [47].

### 2.2. Folate: Its Role in Human Nutrition and Health

Folate, a group of water-soluble micronutrients, is an essential component involved in the biosynthesis of deoxyribonucleic acid (DNA) [48]. It is commonly referred to as vitamin B9 and should not be confused with folinic acid or leucovorin, which is more technically identified as 5-formyltetrahydrofolate (5-FTHF) [48]. Folate serves as a coenzyme or cosubstrate in single-carbon transfers, playing a crucial role in the synthesis of nucleic acids and the metabolism of amino acids [49,50]. One of its key functions is facilitating the conversion of homocysteine to methionine, a vital step in the synthesis of S-adenosyl-methionine (SAMe), a crucial methyl donor. Another important folate-dependent reaction involves the methylation of deoxyuridylate to thymidylate during DNA formation, which is essential for proper cell division [51]. Folic acid, found in fortified foods and many dietary supplements, represents the fully oxidized monoglutamate form of the vitamin. Additionally, certain dietary supplements may contain folate in the monoglutamyl form, known as 5-MTHF (alternatively L-5-MTHF, 5-methyl-folate, L-methylfolate, or methylfolate) [48]. Folate is converted into tetrahydrofolic acid (THF), which undergoes various transfer and methylation reactions crucial for the synthesis of nitrogenous bases in DNA and ribonucleic acid (RNA), as well as the maturation of red blood cells (RBCs) [51]. The liver and kidney maintain small reserve pools of folate. Being an essential nutrient, folate is not synthesized by humans or other animals, necessitating regular ingestion through dietary sources. Natural sources of folate include leafy green vegetables such as spinach and broccoli, lettuce, liver, eggs, and milk [51]. However, despite the importance of maintaining adequate folate levels, daily intake often falls below the recommended dosage suggested by national health authorities [52], which is currently 400 mcg dietary folate equivalents for nonpregnant adults aged ≥19 years [51]. The estimated total body content of folate is approximately 15 to 30 mg, with approximately half of this quantity stored in the liver, while the remaining portion is distributed among the blood and various body tissues [49]. Serum folate concentrations are frequently employed as an indicator of folate status. A serum folate value above 3 ng/mL is considered indicative of adequate folate levels [49,50,53]. Although serum folate concentrations are commonly used as an indicator of folate status, it is important to note that this measure can be influenced by recent dietary intake and may not accurately reflect long-term status. For a more comprehensive assessment of folate intake over a longer duration, erythrocyte folate concentrations are considered a reliable measure. Adequate folate status is typically indicated by erythrocyte folate concentrations above 140 ng/mL [50,53,54,55].

The development of the central nervous system (CNS) is a critical aspect where folate supplementation plays a significant role. Women who are planning to become pregnant are advised to take folic acid supplements to lower the risk of neural tube defects (NTDs) in the developing foetus, including conditions like spina bifida [52]. It has been suggested that the absence of folate may lead to the increased ubiquitination of genes related to neural tube closure, subsequently impacting their expression and contributing to the formation of NTDs [56]. The fourth week of development is considered a particularly critical period of vulnerability, often before a woman realizes she is pregnant. Therefore, it is recommended to initiate supplementation 5 to 6 months before planning to conceive to ensure sufficient time for the body to achieve the necessary folate levels [57]. Folic acid supplementation has also been linked to a reduced risk of preterm birth [58].

### 2.3. The Link between Folate and Depressive Disorders

Numerous studies have provided evidence suggesting a potential association between folate deficiency and depression [22,59,60,61,62,63]. These findings indicate that folate deficiency is linked to an elevated risk of developing depression, as well as more severe depressive symptoms, longer episodes of depression, and an increased likelihood of depressive symptom relapse [22,30,61,64,65]. The primary neurobiological theory underlying MDD is based on the involvement of monoamine neurotransmitters such as serotonin (5HT), norepinephrine (NE), and dopamine (DA) [66]. These neurotransmitters operate within specific neural circuitries in the brain, which play a role in the development of depressive symptoms [67]. Tetrahydrobiopterin (BH4) is another important cofactor involved in neurotransmitter synthesis. BH4 is required for the synthesis of several neurotransmitters, including 5HT, DA, and nitric oxide. Folate plays a role in BH4 synthesis by contributing to the regeneration of BH4 from its oxidized form [68,69,70,71,72]. When folate levels are low, it might result in reduced levels of DA, NE, and 5HT, thereby creating a neurochemical susceptibility to depression [66]. However, the validity of this rather simplistic model lacks sufficient support from both animal models and human research, necessitating further investigation to establish its accuracy and reliability.

**Figure 1:**
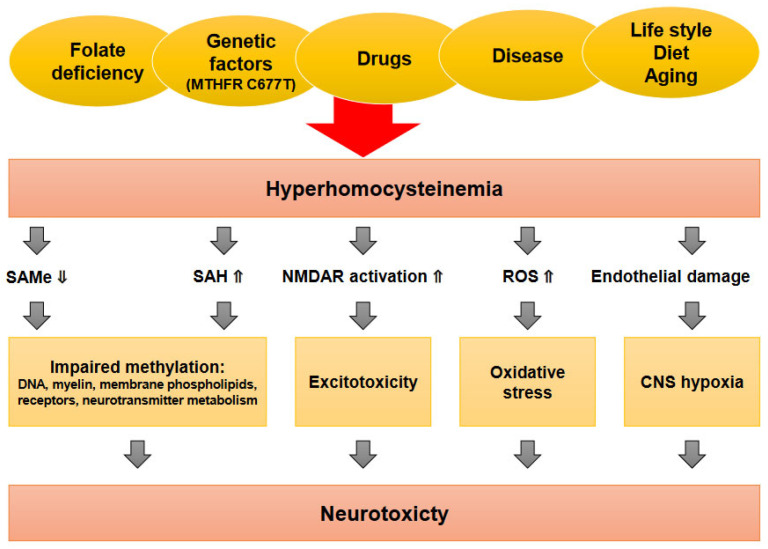
Mechanisms of homocysteine-induced neurotoxicity. The figure depicts the various mechanisms involved in the neurotoxic effects of elevated homocysteine levels. Multiple factors, including nutritive folate deficiency, diseases, aging, and MTHFR C677T polymorphism, can contribute to hyperhomocysteinemia. Elevated homocysteine levels disrupt the delicate balance of key biochemical processes within the central nervous system. The cascade of events triggered by hyperhomocysteinemia includes a decrease in S-adenosylmethionine (SAMe) levels and an increase in S-adenosylhomocysteine (SAH) levels. This imbalance leads to heightened oxidative stress due to elevated reactive oxygen species (ROS) production. The resulting oxidative stress and endothelial dysfunction contribute to neurotoxicity. The neurotoxic effects of elevated homocysteine levels are implicated in the development of depressive illness. The disrupted biochemical pathways and increased oxidative stress ultimately contribute to the pathogenesis of depressive symptoms. The figure has been modified based on [78].

Folate deficiency, in conjunction with other factors such as medications and genetic predisposition, can contribute to hyperhomocysteinemia, characterized by elevated levels of homocysteine [73]. This metabolic imbalance can disrupt the methylation cycle, leading to decreased levels of S-adenosylmethionine (SAMe) and increased levels of S-adenosylhomocysteine (SAH) [74]. One consequence of elevated homocysteine and reduced SAMe is the activation of the N-methyl-D-aspartate (NMDA) receptors, which play a role in excitatory neurotransmission. Excessive NMDA receptor activation can disrupt the delicate balance of neurotransmitter systems, contributing to neuronal excitotoxicity and oxidative stress [75,76]. The impaired endothelial function and increased reactive oxygen species (ROS) production associated with hyperhomocysteinemia can also have detrimental effects on vascular health and integrity [77]. Endothelial dysfunction can compromise blood flow and nutrient delivery to the brain, further exacerbating neuronal damage and mood disturbances. The dysregulation of these biochemical processes can have cascading effects on neurophysiology and mood regulation (Figure 1) [73,78].

Research in genetics has implicated a specific genetic variation in the methyltetrahydrofolate reductase (MTHFR) enzyme, known as the C677T TT genotype, in depression. This particular genotype affects the enzyme responsible for folate metabolism. Meta-analytic evidence has indicated that individuals with the TT genotype are approximately 1.37 times more likely to have a diagnosis of depression compared to those with the CC genotype [79]. In a study by Lok et al., the relationship between early-life adversity, MTHFR genotype, and the recurrence of MDD was investigated. The researchers followed patients with a history of recurrent depression currently in remission for 5.5 years to observe depression recurrence. The study found that the presence of the MTHFR T allele, combined with early-life adversity, was a significant predictor of depression recurrence. The severity of depression was highest in individuals with the TT genotype and lowest in those with the CC genotype. The severity of depression also correlated with the number and severity of childhood traumatic events [80]. It is worth noting that specific ethnic groups have a higher risk for less-functional forms of MTHFR, with the TT genotype being present in up to 10% of Caucasians and up to 22% of Hispanic or Mediterranean populations [81]. Since MTHFR is responsible for converting folate into its active form, L-methylfolate, individuals with genetic polymorphisms affecting this conversion may benefit from the administration of L-methylfolate rather than folic acid. By using L-methylfolate, the defective enzyme can be bypassed, potentially addressing the folate-related deficiencies more effectively in this population [82,83]. Future research endeavours should focus on investigating in greater detail how MTHFR polymorphisms contribute to the susceptibility of depression. Epigenetic modifications, including DNA methylation and histone modifications, can regulate gene expression patterns without altering the underlying genetic sequence. Investigating the epigenetic mechanisms involved in modulating MTHFR expression can shed light on how environmental factors, such as adverse childhood experiences and nutrition, interact with genetic predispositions to shape an individual’s susceptibility to depression [84,85].

### 2.4. The Potential of Folic Acid as a Neuro-Nutraceutical for Depression

Emerging research suggests that the functioning of the one-carbon cycle may have implications for the response to antidepressant treatment. Deficiencies in any of the components within the cycle have been independently linked to mental illness [86]. However, among these components, folate levels have received particular attention due to their widespread prevalence in depression [69]. L-methylfolate is the active form of vitamin B9. It is the only form able to cross the blood–brain barrier [87]. Research has demonstrated that individuals with folate deficiency may exhibit an inadequate response to antidepressant medications, which can contribute to the characterization of their condition as “treatment-resistant” depression [88]. Both folic acid and L-methylfolate have demonstrated efficacy in individuals with depression, either as a standalone treatments or as supplementary therapies [89,90]. For example, in an RCT conducted by Reynolds et al., a monotherapy with L-methylfolate showed comparable effectiveness to a standard antidepressant in the treatment of mild-to-moderate depression [91]. The study included 19 patients treated with L-methylfolate (25 mg of active L-methylfolate per day) and 20 patients treated with amitriptyline (150 mg/day) for a duration of 6 weeks. The response rates were 42% for L-methylfolate and 35% for amitriptyline. Notably, no side effects were reported with L-methylfolate, while three patients had to withdraw from the study due to unacceptable side effects from amitriptyline. Additionally, a noteworthy observation was the correlation between the antidepressant response to L-methylfolate and the increase in RBC folate levels [91]. However, studies comparing folate or methylfolate with placebo as a monotherapy for various conditions are scarce, and the current evidence suggests that these compounds may be more effective as adjunct treatments rather than standalone options [92]. Nevertheless, such studies highlight the significance of evaluating the folate status of patients who exhibit signs of being “treatment resistant” [69,88,93,94]. The U.S. Food and Drug Administration (FDA) has granted licensure to L-methylfolate as a medical food due to its demonstrated efficacy in addressing folate deficiencies and its potential impact on depressive symptoms.

The findings of many studies investigating the efficacy of folate in the context of depression are constrained by their relatively short duration. L-methylfolate was explored as an adjunctive therapy within a long-term double-blind, placebo-controlled trial [95]. In this study, 24 patients diagnosed with MDD and exhibiting RBC folate deficiency were enrolled. The patients received 15 mg per day of L-methylfolate as an addition to their ongoing antidepressant treatment. The trial lasted for a period from 3 to 6 months. Patients who received L-methylfolate as an adjunctive therapy experienced significantly greater improvements compared to those who were given a placebo [95]. A recent meta-analysis examining the use of folate supplementation in the treatment of MDD reached nuanced conclusions. According to the authors, while the inactive form of folate, folic acid, did not show substantial evidence for recommendation, the active forms, L-methylfolate and SAMe, were found to be potentially beneficial as adjunctive treatments for MDD [96]. Furthermore, individuals who are MTHFR C677 TT genotype carriers, resulting in a decreased conversion of folate to its active form, may particularly benefit from L-methylfolate therapy [97]. The World Federation of Societies of Biological Psychiatry (WFSBP) and the Canadian Network for Mood and Anxiety Disorders (CANMAT) guidelines on the use of nutraceuticals in psychiatric disorders suggest that L-methylfolate at a daily dose of 15 mg is provisionally recommended as an adjunctive treatment for MDD. However, folic acid is not recommended for adjunctive use in MDD according to these guidelines [32]. The results of another recent meta-analysis indicated that participants who received the combination of vitamin B9 and SSRI/SNRI treatment showed a 36% increase in the response rate compared to those receiving SSRI/SNRI monotherapy. The calculated number needed to treat (NNT) was 5 [89]. These findings suggest that personalized treatment approaches that consider genetic variations and target folate deficiencies could potentially optimize treatment outcomes in individuals with treatment-resistant depression [89,98]. While RCTs are considered the gold standard for evaluating treatment efficacy, real-world naturalistic studies provide complementary evidence that is essential for translating research findings into clinical practice. The combination of both study designs can provide a more comprehensive and nuanced understanding of interventions, their outcomes, and their applicability in real-world settings [99]. A study conducted by Shelton et al. aimed to assess changes in depression severity and medication satisfaction in patients prescribed L-methylfolate (7.5 or 15 mg per day) in a real-world setting [87]. Patients with MDD were surveyed before and after three months of L-methylfolate treatment. The results showed that patients reported a significant reduction in depression severity scores, with 67.9% of participants responding to treatment and 45.7% achieving remission after an average of 95 days of therapy. Patients also experienced improvements in work/home/social life and expressed higher satisfaction with L-methylfolate compared to their prior medication [87]. These findings suggest that L-methylfolate may be effective in reducing depression symptoms and improving functioning in a naturalistic setting.

The inconsistent and varied results observed in clinical studies regarding the antidepressant effects of folate and structurally related metabolites in individuals with depression highlight the need for further comprehensive investigations [31,32,100]. One of the numerous unanswered questions regarding folate treatment in depression is determining the optimal duration of treatment. There is limited support for short-term supplementation compared to long-term supplementation. Whether folate supplementation should be recommended regardless of folate levels or specifically in cases of folate deficiency only is still a subject of debate and requires further investigation. Although folate supplementation is generally considered safe, it should be approached with caution as a drug-like signaling agent. Like any medication, folate supplementation may have side effects and potential interactions with other psychiatric medications. However, the investigation of these potential side effects and interactions remains underexplored. In a study evaluating the combination of quetiapine and lamotrigine versus quetiapine alone in bipolar depression, an unexpected interaction was observed between lamotrigine and folic acid. It appeared that folic acid supplementation was associated with a diminished response to lamotrigine during the initial 12 weeks of treatment [101]. While further evidence is needed to fully understand this interaction, it is important for clinicians to be aware that folic acid supplementation may potentially reduce the effectiveness of certain psychotropic medications. Careful consideration and monitoring are necessary when combining folic acid with these medications to ensure optimal treatment outcomes.

### 2.5. Exploring Folate and New Therapeutic Avenues in the Treatment of Depression

In the current biomedical understanding of depression, the condition is conceptualized as a disorder that impacts intricate neural networks and involves changes in multiple brain regions and neurotransmitter systems. Antidepressant medications are thought to exert their therapeutic effects by promoting synaptic plasticity, thereby contributing to the amelioration of depressive symptoms [67,102,103,104]. The monoamine hypothesis of depression, which suggests that imbalances in neurotransmitters like 5HT, NE, and DA are responsible for the development of depression, has been a prominent framework in psychiatric research and treatment. However, it has become evident that this hypothesis alone is insufficient in explaining the wide variation in treatment response and providing effective therapy for all individuals with depression [105]. There is a recognized need for the development, testing, and understanding of new agents or treatment modalities for depression that offer improved characteristics compared to existing antidepressants. These characteristics include a more rapid onset of action, better tolerability, and potentially greater effectiveness, especially for individuals who have not responded well to current antidepressant medications [104].

### 2.6. N-Methyl-D-Aspartate Receptor Modulation

Glutamate (GLU) is the most prevalent excitatory neurotransmitter in the brain, and its levels can be elevated by chronic stress [106]. Disturbances in GLU functioning have been observed in brain regions associated with depression [107]. This increase in GLU can disrupt synaptic connectivity and lead to deficits in the functioning of γ-aminobutyric acid (GABA), which is the brain’s primary inhibitory neurotransmitter. The interplay between GLU and GABA, as well as the balance between them in different brain regions, is believed to represent a crucial pathway in depression and a crucial target of action for antidepressant treatments [108]. Research interest in ketamine has surged due to its unique and rapid antidepressant effects. Ketamine is composed of two enantiomers, namely R-ketamine and S-ketamine. Both enantiomers function as anti-glutamatergic agents by acting as antagonists at the NMDA receptor in the brain [109]. The NMDA receptor is a key player in synaptic plasticity and is involved in various neural processes, including learning, memory, and mood regulation. By blocking the NMDA receptor, ketamine modulates GLU signalling and influences the balance between excitatory and inhibitory neurotransmission [110]. Daily supplementation with folic acid has been shown to effectively reduce plasma homocysteine levels by approximately 20% to 30% in individuals with normal to slightly elevated homocysteine levels [111]. Homocysteine is known to act as an NMDA receptor agonist [112]. Homocysteine’s depressogenic effects might be explained via its actions at the NMDA receptor [113]. Reducing homocysteine levels through folic acid supplementation is believed to be one mechanism by which folic acid exerts its beneficial effects on depression [114]. While there are no specific studies examining the synergistic effects of ketamine and folate, it is plausible that combining these interventions could potentially yield synergistic effects [115].

### 2.7. Anti-Inflammatory Agents

**Figure 2:**
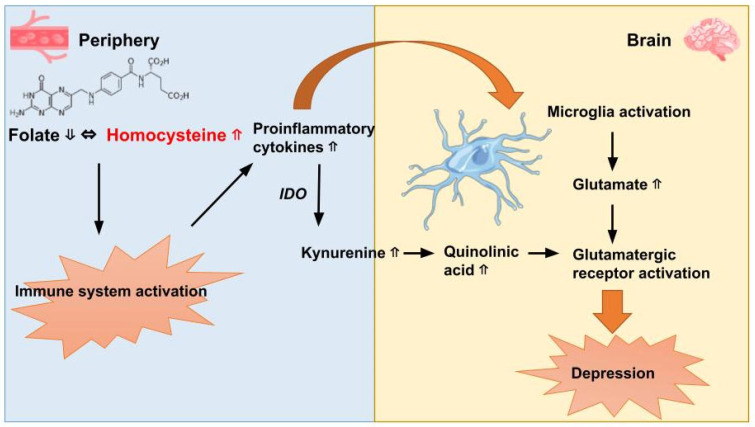
Pathophysiology of inflammation-induced depression linked to folate deficiency. The figure illustrates the cascade of events involved in inflammation-induced depression associated with folate deficiency. Inflammatory stimuli trigger the production of proinflammatory cytokines, which activate various enzymatic processes. One such process is the conversion of tryptophan to kynurenine catalyzed by indoleamine 2,3 dioxygenase (IDO). Cytokine signaling to the brain stimulates microglia activation, resulting in the production of inflammatory mediators. Simultaneously, kynurenine is transported into the brain where it is further metabolized into neurotoxic compounds, including quinolinic acid. Activated microglia also release glutamate, a key excitatory neurotransmitter. Both glutamate and quinolinic acid contribute to the enhancement of glutamatergic neurotransmission, ultimately leading to the development of depressive symptoms.

The notion that immune dysfunction, particularly chronic low-level inflammation, could play a role in depression and serve as a potential treatment target was initially proposed in the early 20th century. This concept emerged from observations made in German asylums, where some patients with depression experienced improvements in symptoms following treatment with vaccine therapy for typhus [116]. Elevated levels of inflammatory cytokines, such as interleukin 6 (IL-6), tumour necrosis factor α (TNFα), and C-reactive protein (CRP), have consistently been found in patients with depression compared to nondepressed control individuals [117]. The relationship between inflammation and depression has been supported by experimental studies that have induced inflammation in rodents, resulting in the emergence of depressive-like symptoms and lethargy [118]. Mendelian randomization studies investigating IL-6 have also provided evidence supporting a causal relationship between inflammation and depression in humans [119]. There are several mechanisms through which inflammation may contribute to depression: Early-life stress and environmental stressors have been shown to increase the levels of pro-inflammatory cytokines, especially in male individuals [120]. These cytokines can act on second messengers such as prostaglandins, activate microglia (the immune cells in the brain), and impact various pathways, including the glutamatergic pathway and the tryptophan and kynurenine system (Figure 2) [121,122,123]. These pathways play important roles in neurotransmitter regulation and neuronal function, and their dysregulation may contribute to the development and progression of depression [124,125]. Moreover, cerebral inflammation has been causally linked to GLU-mediated excitotoxicity [126]. Bai et al. conducted a systematic review and meta-analysis, comprising a total sample size of 1610 participants, to evaluate the efficacy and safety of anti-inflammatory agents in the treatment of MDD [127]. Their findings indicate that various anti-inflammatory treatments, including celecoxib (a cyclooxygenase-2 inhibitor), other non-steroidal anti-inflammatory agents, omega-3 fatty acids, and statins, demonstrated a beneficial effect in reducing depressive symptoms. These treatments were found to be effective when used as a monotherapy or as an adjunct to antidepressant medication [127].

Optimal folate levels have been associated with prevention of endothelial dysfunction in inflammatory diseases, and folate has the potential to modulate inflammatory responses through DNA methylation and synthesis processes [128]. Chronic inflammatory diseases are often associated with low folate levels, suggesting a possible role of inadequate folate supply in their development or the increased folate requirements due to chronic inflammation [129]. These findings provide a direct connection with studies on the inflammatory role of homocysteine, demonstrating that homocysteine can induce endothelial injury, DNA dysfunction, smooth muscle cell proliferation, oxidative stress, reduced glutathione peroxidase activity, impaired nitric oxide synthase function, and inflammation [130]. The systematic review by Asbaghi et al. included twelve RCTs and investigated the effects of folic acid supplementation on inflammatory markers [131]. The analysis showed that folic acid supplementation significantly reduced serum concentrations of CRP. However, folic acid supplementation did not have significant effects on serum concentrations of IL-6 or TNF-α. The dose–response analysis indicated that higher dosages of folic acid supplementation were associated with lower CRP concentrations. Overall, the findings suggest that folic acid supplementation may have anti-inflammatory benefits by reducing CRP levels, but further research with larger and more diverse populations is needed to validate and expand upon these findings [131].

There is a growing interest in potential anti-inflammatory treatments for depression. In addition, recent studies have provided compelling evidence indicating that the anti-inflammatory effects of ketamine and electroconvulsive therapy (ECT) contribute to their efficacy in treating treatment-resistant depression [132,133]. Additional research is required to obtain a comprehensive understanding of the intricate interplay between folate and inflammation, as well as to ascertain the potential implications of this relationship in the treatment of depression. By investigating the mechanisms through which folate influences inflammatory processes, such as modulating immune responses or regulating pro-inflammatory cytokines, researchers can potentially uncover novel therapeutic strategies for managing depression.

### 2.8. Gut–Brain Axis

Recent research has indicated a potential association between the microbiome and depression [134]. The microbiome encompasses the wide-ranging assembly of microorganisms that inhabit the human body, predominantly within the gastrointestinal tract, along with their combined genetic material [135]. The human colon contains a diverse and abundant microbial community, with approximately 10^11^ microorganisms per gram of intestinal content. The majority of these microorganisms are anaerobic bacteria. In addition to bacteria, the microbiota of the colon also encompasses archaea, yeasts, and other eukaryotes. Together, they form a complex ecosystem within the gastrointestinal tract. The gut microbiome consists of the combined genetic material of approximately 100 trillion microorganisms that reside in the gastrointestinal tract. These microorganisms possess a gene repertoire that is approximately 150 times larger than the human genome [136]. While there is growing evidence suggesting a link between alterations in the microbiome and depressive disorders [137,138,139,140,141,142,143], it is important to note that currently most findings are correlational, and causative relationships have not been firmly established yet [144]. The exact mechanisms through which the microbiome may influence mental health are unclear, and further research is needed to determine the precise role of the microbiome in depression and other behavioral disorders [145]. Nonetheless, the emerging evidence highlights the potential significance of the gut–brain axis and the interplay between the microbiome and mental health, providing new avenues for exploration in understanding and treating depressive disorders [146,147,148,149].

The microbial community in the gut possesses enzymes and metabolic capabilities that the host lacks, allowing for the breakdown and utilization of complex molecules such as dietary fibres and other indigestible components. The colon provides an environment where microbial communities can synthesize and release various vitamins, such as vitamin K, biotin, pantothenic acid, and certain B vitamins. Through these processes, the gut microbiota contributes to the overall nutrient balance and energy metabolism of the host [150,151]. Unlike dietary vitamins, which are primarily absorbed in the proximal part of the small intestine, the absorption of microbial vitamins takes place predominantly in the colon. Colonocytes, the cells lining the colon, have been observed to possess the ability to absorb certain vitamins produced by the gut microbiota. These include biotin, thiamine, folates, riboflavin, pantothenic acid, and menaquinones [152,153]. The absorption of these microbiota-produced vitamins by colonocytes underscores the intricate relationship between the gut microbiota and the host, highlighting the potential influence of microbial activities on the vitamin status and cellular functions within the colon.

The colonic microbiota has the capability to synthesize folate, and there is potential for its absorption across the colon [154]. However, the precise contribution of colonic folate synthesis to the human host’s overall folate status remains uncertain [155]. Nevertheless, both human and animal studies indicate that the abundance and structure of folate (specifically its monoglutamylated forms) in the large intestine are substantial enough to potentially impact folate status and potentially essential downstream folate-dependent functions [156,157]. Enhancing colonic folate production in depressed individuals could potentially improve their folate status and support optimal mental health. There are several potential ways to improve colonic folate production in depressed individuals, one being probiotic supplementation. Probiotics, which are live microorganisms that confer health benefits when consumed in adequate amounts, have been studied for their potential to modulate the gut microbiota and improve mental health outcomes [158]. Introducing specific probiotic strains that have the ability to produce folate into the gut microbiota could increase folate levels. This concept has been supported by a proof-of-principle study conducted on Wistar rats, demonstrating that administering folate-producing Bifidobacteria resulted in an improvement in host folate status [156]. Lactobacillus strains, such as *L. rhamnosus* and *L. plantarum*, have been investigated for their folate-producing capabilities [159]. Lactobacillus strains isolated from the human gastrointestinal tract have shown promise as probiotics capable of producing folate. Similarly, strains obtained from fermented foods have been explored as microbial starters for the production of folate-enriched dairy products, thus enhancing their nutritional value. In this regard, studies have been conducted to examine the vitamin needs of lactobacilli and to evaluate the impact of their growth in different media on folate levels. These efforts aim to harness the folate-producing capabilities of lactobacilli for beneficial applications in the food and probiotics industries [160,161,162,163,164]. Clinical studies are necessary to validate the use of these folate-producing probiotic interventions and to determine their potential benefits in treating depression. It is crucial to verify the viability of these strains within the human gut and their capacity to produce folate in the colonic environment. As many probiotic interventions lack a clear mechanistic rationale for their clinical application [165], conducting such studies would be of significant interest as they would provide rational and data-driven probiotic applications based on empirical evidence. These clinical studies would help bridge the gap between preclinical research and practical implementation, offering valuable insights into the potential therapeutic role of folate-producing bacterial consortia in the management of depression.

### 2.9. The Protective Effects of Folic Acid against Suicide

Suicide is a pressing public health concern that affects individuals of all ages, genders, and geographical regions worldwide [166]. Every case represents a tragic loss, emphasizing the urgent need for prevention efforts [166]. Psychiatric disorders play a significant role in the majority of suicides and suicide attempts, with numbers reported to be at least 10 times higher than in the general population. The percentage of completed suicides within the context of psychiatric illness varies, ranging from 60% to 98% of all suicides [167]. Among the reported 5.2 million deaths in the European Union (EU) in 2015, intentional self-harm accounted for 56,200 cases (1.1% of total deaths). The majority of suicides, approximately 77%, involved men, while around 31% occurred among individuals aged between 45 and 60 years [168]. In general, the age-standardized suicide rate at a global level has shown a modest decline. However, it is important to note that this trend is not universally observed in all countries worldwide. If the current rate of decline persists, it is unlikely that global targets for reducing suicide mortality will be achieved [166]. This highlights the need for sustained and targeted efforts to address and prevent suicides on a global scale. The significance of biologic interventions is frequently undervalued within suicide prevention strategies [169]. Lithium and clozapine have been extensively studied and have shown consistent evidence of their ability to reduce the risk of suicide in specific clinical populations [170]. While lithium and clozapine have demonstrated efficacy in reducing suicide risk, they may not be suitable or accessible for all individuals at risk. Indeed, the pursuit of safer and more cost-effective biological alternatives for suicide prevention is highly desirable.

Individuals who are at risk of future suicide have been found to have low levels of folate in both their serum and RBCs [171]. In a recent study conducted in South Korea, a significant association was discovered between serum folate levels and both fatal and nonfatal suicide attempts during the follow-up period [172]. The study reported an area under the curve (AUC) value of 0.77, indicating a moderate predictive ability. By using a cutoff point of 6 ng/mL, the researchers found an adjusted odds ratio (OR) of 2.69 (95% CI, 1.27–5.69) for dichotomized serum folate levels. Additionally, for individuals classified as having folate deficiency, defined as serum folate levels below 3 ng/mL, the OR was 2.84 (95% CI, 1.19–6.77) [172]. Gibbons and colleagues devised a high-dimensional method called iDEAS for identifying signals in pharmacoepidemiology. Through an atheoretical approach, they unexpectedly discovered an anti-suicidal signal among individuals who were using folic acid supplements [173]. The researchers conducted a more rigorous pharmacoepidemiological follow-up study to validate their data mining discovery [174]. They employed a within-person exposure-only cohort design to examine the dynamic relationship between folic acid prescription fills over a 24-month period and incidents of suicide attempts and intentional self-harm. The study utilized data from the MarketScan pharmacoepidemiologic database, which comprises US medical claims of privately insured patients who received a folic acid prescription between 2012 and 2017. To establish a control, the same analysis was repeated using a different supplement (cyanocobalamin, vitamin B12). The analysis was adjusted for various factors, including age, sex, diagnoses related to suicidal behavior, diagnoses related to folic acid deficiency, folate-reducing medications, history of folate-reducing medications, and history of suicidal events. The hazard ratio (HR) for folic acid in relation to suicide events was 0.56 (95% CI, 0.48–0.65). Similar results were obtained for a typical dosage of 1 mg of folic acid per day (HR, 0.57; 95% CI, 0.48–0.69) and among women of childbearing age (HR, 0.60; 95% CI, 0.50–0.73). Additionally, a duration–response analysis (for the 1 mg dosage) revealed a 5% reduction in suicidal events per month of additional treatment (HR, 0.95; 95% CI, 0.93–0.97) [174]. In an RCT involving 475 patients, the participants were randomly assigned to receive either 5 mg of folic acid daily (223 patients) or a placebo (217 patients) alongside their antidepressant treatment for a duration of 12 weeks. The study did not find a significant difference between the two groups regarding suicide ratings (Mini International Neuropsychiatric Interview (MINI) suicidality subscale) [175]. However, it is worth noting that the observation period of 12 weeks might have been too short to detect an effect on the specific endpoint of suicide and that suicidality ratings might not reliably predict actual suicidal events [176].

The positive results obtained by Gibbons and colleagues, coupled with the wide availability, relatively low cost, and favorable safety profile of folic acid, warrant further large-scale and long-term investigations into its potential for suicide prevention.

### 2.10. Population Suicide Risk: Exploring Latitude Variation, Genetic Influences, Diet, and Folate Status

The global incidence of suicide demonstrates variability based on geographical latitude. The calculated average suicide prevalence was 8.12 (range: 6.77–9.47) within the latitude range of 0–14°, 8.54 (range: 2.92–14.15) for 15–29°, 9.97 (range: 6.29–13.65) across 30–44°, 19.23 (range: 16.67–21.80) for 45–59°, and 15.28 (range: 9.12–21.44) within 60–75°. A regression analysis revealed a beta coefficient of 0.255, signifying that a 1° increment was associated with a 0.255 per 100,000 people rise in suicide prevalence. An et al. conducted a meta-analysis indicating that their regression model incorporating latitude change accounted for approximately 27.3% of the suicide prevalence (adjusted R-squared: 0.273) [177]. The observed variation cannot be adequately accounted for solely by disparities in socioeconomic outcomes [178]. The proposed “Finno-Ugrian suicide hypothesis” highlights that European regions with prevalent Finno-Ugrian ethnicities, languages, or cultural influences exhibit elevated suicide rates [179]. This hypothesis suggests that population distinctions in genetic risk factors could contribute to the geographical diversity in European suicide rates. In particular, the hypothesis posits a potential interplay among genetic susceptibility, alcohol exposure, and tendencies toward suicidal behavior [179]. Despite encountering skepticism on several occasions since its inception [180], the Finno-Ugric suicide hypothesis has persistently garnered support over the years. Authors have consistently presented substantiating evidence that reinforces its credibility [181]. The potential contributions of diet and the gut microbiota to geographical variations in mental health and suicide risk have been overlooked. Diet and gut microbiota composition exhibit variations based on geographical location [182,183]. Traditional diets such as the Okinawan diet have been associated with greater nutrient density and generally more favorable health outcomes [184]. Evidence indicates that individuals with a history of suicide attempts exhibited significant under-consumption of fruits, vegetables, and meat [185]. Following dietary patterns aligned with the traditional Japanese diet, characterized by an abundant consumption of vegetables, fruits, grains, legumes, fish, and foods rich in polyunsaturated fatty acids (PUFA) and dietary fiber, is linked to lowered risks of depression and suicide, both within Japan and on a global scale [186,187]. The traditional Chinese diet prominently features a substantial amount of green vegetables, consequently providing a rich source of folate [27]. Utilizing red blood cell folate measurements, the Chinese province of Shaanxi displayed a 15% prevalence of folate insufficiency among women of childbearing age [188]. In comparison, this rate was only 2.5% in Taiwan [189]. In multiple Western high-income countries, the rate of red blood cell folate insufficiency was consistently higher [190]. The observed range spanned from 22% (in Canada) [191] to a concerning 100% (in Sweden) [192]. In Taiwan, where the Chinese diet is commonly embraced, there is a strikingly low lifetime prevalence of major depression at 1.2% [193], which is in contrast to a substantial 15% observed in the German National Cohort (NAKO), for instance [194]. However, this contrast is not always mirrored in suicide rates. For instance, in 2022, Taiwan recorded 3787 deaths attributed to intentional self-harm, translating to just over 16 deaths per 100,000 residents [195]. In contrast, within Germany, the suicide rate fluctuated across federal states, ranging from 7.4 to 16.1 per 100,000 inhabitants in 2021 [196]. On a broader scale, the European Union exhibited an average suicide rate of 11 deaths per 100,000 inhabitants in the year 2015 [168]. This underscores the intricate nature of suicidality, which emerges as a complex phenomenon characterized by a multitude of underlying factors rather than a singular or a few clearly defined causes. Suicidal tendencies are intertwined with a web of intricate interactions among genetics, environment, socio-cultural elements, and psychological dynamics [197]. However, while considering this complexity, the realm of dietary variation warrants greater attention within research. Exploring whether variations in dietary folate supply correspond with the risk of self-harm and suicide is an avenue that holds significant promise. Such investigations could potentially shed light on previously unexplored dimensions of the relationship between nutritional intake and mental wellbeing.

### 2.11. Discussion and Prospects for Further Study

Neurometabolic research in the field of neuropsychiatry remains relatively underexplored and warrants greater attention from the psychiatric research community. Rather than solely focusing on the quest for novel drugs with uncertain mechanisms and adverse side effects, there is a need to invest more in the neurometabolic evaluation of major depression [198]. This includes examining the metabolic profiles of both responders and non-responders to treatment. One promising area for such research lies in the investigation of one-carbon metabolism, which holds great potential due to its intimate connection with neurotransmitter synthesis [86]. Folic acid and L-methylfolate have demonstrated effectiveness and good tolerability as treatment options for individuals with depressive disorders, either as adjuncts to antidepressant medications or as a monotherapy. In particular, L-methylfolate may offer specific benefits for patients with the MTHFR C667T TT genotype [199]. Low folate levels are common among individuals with depression, influenced by various factors such as lifestyle, medications, and genetic predisposition. But even individuals with normal folate levels may still experience benefits from folic acid or L-methylfolate supplementation. Folic acid or L-methylfolate augmentation has demonstrated good tolerability, with no established reports of inducing manic episodes or significant drug interactions. However, the potential drug interactions of these nutraceuticals, particularly when combined with antidepressants and other psychotropic medications, require further investigation, as limited research has been conducted in this area. Further research is essential to gain a deeper understanding of how folate interacts with emerging and promising avenues in biological psychiatry. These include the modulation of NMDA receptor signaling, the attenuation of inflammation, and the restoration of gut microbiota eubiosis. Understanding the mechanisms by which the gut microbiota, including probiotics, produce and utilize B vitamins, such as folate, can provide valuable insights into the gut–brain axis and the potential therapeutic applications of probiotics in mental health conditions like depression. However, further research is needed to explore this avenue and establish the clinical efficacy of probiotics and microbial vitamin synthesis in the context of depression treatment.

The inconsistent and mixed results observed in studies investigating the efficacy of folic acid and L-methylfolate in depression treatment have raised concerns [92]. One possible explanation for these varied outcomes is the failure to account for the substantial heterogeneity in the underlying pathogenesis of depression among the included individuals. Depression is a complex and multifaceted disorder with diverse etiological factors and mechanisms involved. A more comprehensive understanding of depression necessitates a systems psychiatry approach that considers the intricate interplay of various biological, psychological, and environmental factors [200]. Moreover, the concept of personalized or precision medicine might be crucial in the context of folate treatment for depression. Each individual with depression may exhibit unique genetic, physiological, and environmental characteristics that influence their response to folate supplementation. By adopting a precision medicine approach, it becomes possible to identify specific factors that predict an individual’s response to folate treatment [201]. This may involve genetic testing, microbiome analysis, an assessment of folate metabolism pathways, or other relevant biomarkers to tailor treatment strategies accordingly. With the declining cost of consumables for high-dimensional next-generation biological data profiling, there is a potential for these techniques to be incorporated into routine clinical practice for large patient populations in the near future [202]. Applying a systems psychiatry framework and incorporating personalized medicine principles can provide a more comprehensive and accurate understanding of the factors contributing to treatment response in depression [203]. It can help to identify subgroups of patients who are more likely to benefit from folate supplementation, optimizing the therapeutic approach for individuals with depression. This approach acknowledges the complexity and heterogeneity of depression, aiming to provide tailored treatments based on an individual’s unique characteristics and needs.

Currently, evidence-based NTD prophylaxis is implemented across over 60 nations worldwide. Mandatory folic acid fortifications, as introduced in the USA and Canada, have led to a reduction of 25% to 45% in NTD pregnancies [204]. Given the pervasive prevalence of depressive disorders and other behavioral health conditions, there is potential for folate acid fortification to serve as a public health strategy in tackling the escalating rates of depression. However, apprehensions regarding an elevated cancer risk associated with excessive folate or folic acid dosages pose challenges to the adoption of such policies [205]. Nevertheless, it is noteworthy that neither the comprehensive global World Cancer Research review nor the report by the European Food Safety Authority in the EU have provided data suggesting an augmented cancer risk linked to physiological folate intake levels [204].

To establish the effectiveness and acceptability of folic acid and L-methylfolate, future research of a rigorous methodological quality is necessary. It is recommended to conduct double-blind RCTs that adhere to stringent standards, including robust randomization procedures, proper allocation concealment, effective blinding techniques, and meticulous handling of missing data [206]. Nevertheless, alternative approaches within randomized controlled trials hold considerable significance. These include non-inferiority trials or study frameworks, wherein the new drug or placebo is administered concurrently with standard antidepressant or anti-manic treatment. Furthermore, the incorporation of inventive statistical methodologies like marginal structural models or Q-learning is paramount. These methodologies aid in assessing potential causal effects linked to diverse fixed-treatment regimens that are implemented in real clinical settings. Such strategies contribute to the formulation of tailored treatment recommendations that align with the unique characteristics of individual patients [207]. Future studies should include individuals of both sexes, aged 18 years and over, from various ethnic backgrounds, and in different care settings (inpatient, outpatient, primary care). Subgroup analyses should be performed for specific populations, such as those with treatment-resistant depression, psychotic features, mixed features, anxiety symptoms, or atypical depression.

## 3. Conclusions

In conclusion, the realm of neurometabolic research within neuropsychiatry remains a promising yet relatively unexplored domain, necessitating greater focus from the psychiatric research community. Instead of solely pursuing novel drugs, investing in the comprehensive neurometabolic assessment of major depression is crucial. The exploration of one-carbon metabolism, linked intimately with neurotransmitter synthesis, holds significant potential. Folic acid and L-methylfolate exhibit promise, particularly for individuals with depression, offering benefits both as adjuncts to antidepressants and as monotherapies. The efficacy, tolerability, and potential interactions of these compounds warrant further investigation, especially when combined with psychotropic medications. Understanding their synergy with emerging biological psychiatry avenues, such as NMDA receptor modulation, inflammation attenuation, and gut–brain axis interaction, is pivotal. Tailoring folate treatment through personalized medicine principles, incorporating genetic and biomarker insights, is a forward-looking approach that recognizes the complexity of depression. Rigorous research through double-blind RCTs, embracing diverse populations and clinical settings, is necessary to establish the efficacy of folic acid and L-methylfolate. This effort can extend to suicide prevention, leveraging folic acid’s safety and affordability. Overall, a comprehensive systems psychiatry perspective combined with personalized medicine holds the key to unraveling depression’s intricacies and optimizing treatment strategies for individuals based on their unique characteristics and needs.
